# Association between serum calcium level and the risk of acute kidney injury in ICU patients with subarachnoid hemorrhage: a retrospective cohort study

**DOI:** 10.3389/fneur.2024.1433653

**Published:** 2024-12-11

**Authors:** Zhenlin Zhao, Kuntai Xiao, Sirong Zhao, Kangfeng Liu, Fu Huang, Hua Xiao

**Affiliations:** ^1^Department of Neurosurgery, The Huadu District People's Hospital of Guangzhou, Guangzhou, Guangdong, China; ^2^Department of Critical Care Medicine, The Huadu District People's Hospital of Guangzhou, Guangzhou, Guangdong, China

**Keywords:** serum calcium, SAH, AKI, MIMIC database, ICU

## Abstract

**Aim:**

This study aimed to evaluate the association between serum calcium level and the risk of acute kidney injury (AKI) in patients with subarachnoid hemorrhage (SAH).

**Methods:**

In this retrospective cohort study, data on adults from the Medical Information Mart for Intensive Care (MIMIC-III and MIMIC-IV) databases, spanning from 2008 to 2019, were extracted. In the logistic regression models, confounding variables, including age, white blood cell (WBC), systolic blood pressure (SBP), heart rate, blood urea nitrogen (BUN), glucose, international normalized ratio (INR), and the Charlson Comorbidity Index (CCI), were finally adjusted by stepwise regression. The outcome event was the occurrence of AKI after intensive care unit (ICU) admission. The univariate and multivariate logistic regression models were utilized to assess the association between serum calcium level and the risk of AKI in SAH patients, with odds ratios (ORs) and 95% confidence intervals (CIs). To further explore the association, subgroup analyses were performed, stratified by age, Glasgow Coma Scale (GCS) scores, drugs, and surgical methods.

**Results:**

A total of 1,128 patients with SAH were included in the study, of which 457 patients developed AKI. Low levels of serum calcium were significantly associated with a high risk of AKI in patients with SAH, with an OR (95%CI) of 1.38 (1.01–1.89). Further subgroup analyses showed that low levels of calcium were significantly associated with a high risk of AKI in SAH patients aged ≥60 years (OR = 0.27, 95%CI: 0.09–0.83), who had GCS score ≥13 (OR = 1.57, 95%CI: 1.08–2.30), who did not use calcium channel blockers (CCB) (OR = 2.22, 95%CI: 1.16–4.25) and angiotensin-converting enzyme (ACE) inhibitors (OR = 1.51, 95%CI: 1.06–2.14), and who did not undergo aneurysm embolization (OR = 1.48, 95%CI: 1.01–2.17) and aneurysm clipping (OR = 1.45, 95%CI: 1.04–2.01).

**Conclusion:**

The results of our study indicated that low levels of serum calcium were significantly associated with the risk of AKI in patients with SAH.

## Introduction

Subarachnoid hemorrhage (SAH) is a devastating condition caused by the rupture of cerebral vessels, with an overall incidence of approximately 50,000–100,000 per year (1) and an overall mortality of approximately 20% (2). Acute kidney injury (AKI) is a common complication in the course of neurocritical care for patients with SAH (3), exhibiting an incidence rate of 4%−25% (4–6). AKI can aggravate the poor prognosis in SAH patients, and the occurrence of AKI may be related to the increase in short-term and long-term mortality rates in SAH patients (7). Therefore, identifying indicators closely related to the risk of developing AKI is essential for the effective management and improved prognosis of patients with SAH.

Electrolyte imbalance is a common phenomenon after SAH, which increases the risk of chronic comorbidities (8–12). Electrolyte levels, such as serum magnesium, calcium, potassium, and sodium, have been reported to serve as predictors of the risk of AKI (13–17). Calcium plays a crucial role in regulating blood pressure, managing oxidative stress, and contracting vascular smooth muscle (18, 19) and is significantly associated with a poor prognosis in patients with intracerebral hemorrhage (ICH) (20). Previous studies have reported a U-shaped association between serum calcium levels and the risk of AKI in hospitalized patients (14). In addition, various studies have found that cardiovascular complications, such as low left ventricular ejection fraction, left ventricular volume load overload, and cardiac arrhythmia, which are related to low levels of serum calcium, may contribute to the development of AKI (19, 21–24). To the best of our knowledge, no studies have investigated the association between serum calcium levels and the risk of AKI in patients with SAH.

Therefore, this study used the data obtained from the Medical Information Mart for Intensive Care (MIMIC-III and MIMIC-IV) databases to analyze the association between serum calcium level and the risk of AKI in SAH patients admitted to the intensive care unit (ICU). To further explore the association, subgroup analyses were performed, stratified by age, Glasgow Coma Scale (GCS) scores, drugs, and surgical methods.

## Methods

### Study design and population

In this retrospective cohort study, patient data were extracted from the Medical Information Mart for Intensive Care [MIMIC-III (https://mimic.mit.edu/docs/iii/) and MIMIC-IV (https://mimic.mit.edu/docs/iv/)] databases (certificate number: 13505748). These publicly available databases contain electronic health records of patients admitted to the intensive care units (ICUs) of the Beth Israel Deaconess Medical Center and the Tertiary Academic Medical Center in Boston, Massachusetts, USA between 2001–2012 and 2008–2019. The project was approved by the Institutional Review Boards of the Beth Israel Deaconess Medical Center (Boston, MA) and the Massachusetts Institute of Technology (Cambridge, MA). As all personal information was de-identified, informed consent from the patients was not required.

The inclusion criteria were as follows: (1) patients aged ≥18 years; (2) patients admitted to the ICU for the first time; (3) patients diagnosed with spontaneous SAH (25); (4) patients with a measurement of serum calcium levels; (5) patients diagnosed with AKI; (6) patients with an ICU stay exceeding 24 h; and (7) patients with a measurement of GCS. The exclusion criteria were as follows: (1) patients receiving renal replacement therapy (RRT); (2) patients diagnosed with end-stage renal disease (ESRD) (26, 27); and (3) patients who develop AKI within 24 h of admission.

### Data extraction and definitions

In this study, the following variable information was collected: age, gender, race, vital signs [heart rate, temperature, respiratory rate, systolic blood pressure (SBP), and diastolic blood pressure (DBP)], sequential organ failure assessment score (SOFA), simplified acute physiology score II (SAPS II), Glasgow Coma Scale (GCS) score, Charlson Comorbidity Index (CCI) score, laboratory tests [estimated glomerular filtration rate (eGFR), white blood cell (WBC), red blood cell distribution width (RDW), hematocrit, bicarbonate, blood urea nitrogen (BUN), glucose, international normalized ratio (INR), chloride sodium, magnesium, potassium, and calcium], and medication use and therapy [aneurysm clipping, aneurysm embolization, calcium channel blockers (CCB), angiotensin-converting enzyme (ACE) inhibitors, mannitol use, and osmolar contrast use].

The definition of AKI and serum calcium is as follows:

The occurrence of AKI was determined based on the criteria proposed by the Kidney Disease Improving Global Outcomes (KDIGO) (28). AKI was diagnosed if there was an increase in serum creatinine (SCr) by ≥0.3 mg/dl within 48 h, an increase in SCr by ≥1.5 times of the baseline value within 7 days, or a urine volume of <0.5 mL/kg/h for 6 h.

The total serum calcium levels at admission were categorized into the following tertiles: <2.0709, 2.0709–2.2206, and >2.2206 mEq/L. A calcium level of 2.0709–2.2206 mEq/L was selected as the reference group for comparing the outcome because it indicated a U-shaped relationship between serum calcium level and the risk of acute kidney injury (AKI) in hospitalized patients (14).

### Outcome and follow-up

The outcome event in this cohort was the occurrence of AKI after ICU admission. The start time of the follow-up was the time of first admission to the ICU, and the end time of the follow-up was the time of discharge or the occurrence of AKI.

### Statistical analysis

Continuous variables with a normal distribution were presented as mean ± standard deviation (SD), and Student's *t*-test was conducted to test the differences between the groups. Non-normally distributed continuous variables were presented as medians and quartiles [M (Q1, Q3)] and compared using the Mann–Whitney U-test. Categorical variables were represented as numbers (n) and percentages, and the chi-squared test was conducted for comparison between the groups.

Imputation was performed for missing variables. Sensitivity analyses were performed on the data before and after the imputation (Supplementary Table S1). The variables that were significantly different (with *p* < 0.05) between the non-AKI group and the AKI group were considered potential confounding factors and included in the selection process. Then, a bidirectional stepwise regression method was utilized to select the variables that were significantly associated with AKI (with *p* < 0.05). Finally, the selected covariates were included in the adjustment of the multivariate model (namely model 2) (Supplementary Table S2). Logistic regression analyses were conducted to explore the association between serum calcium level and the risk of AKI in SAH patients, and odds ratios (ORs) with 95% confidence intervals (CIs) were calculated. A restricted cubic spline (RCS) curve was drawn to reflect the risk of AKI along with the serum calcium change in patients with SAH. In addition, subgroup analyses were performed, stratified by age (<60 years or ≥60 years), GCS scores (<13 or ≥13) (29), drugs (calcium channel blockers and ACE inhibitors), and surgical methods (aneurysm embolization and aneurysm clipping).

Data cleaning, missing value imputation, and modeling were performed using R version 4.2.2 (Institute for Statistics and Mathematics, Vienna, Austria). Statistical analysis and sensitivity analyses were performed using SAS 9.4 software (SAS Institute Inc., Cary, NC, USA). A *p*-value of <0.05 was considered significant for all analyses.

## Results

### Baseline characteristics of patients

**Figure 1:**
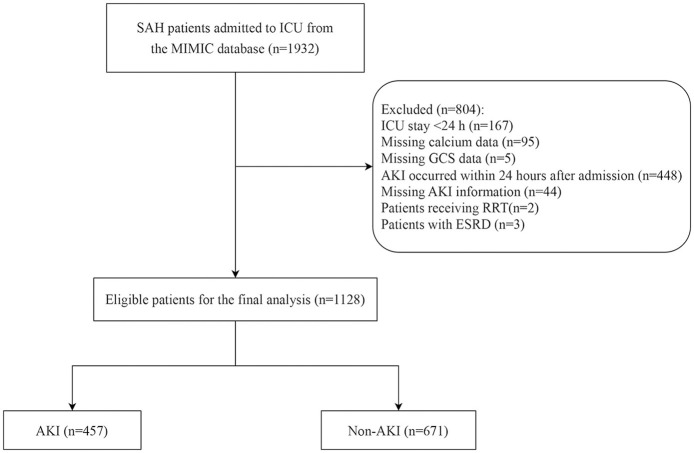
Subject screening flowchart.

Figure 1 shows the selection process of the participants. A total of 1,128 patients with SAH were included in the analysis, of which 457 patients developed AKI. Table 1 shows the comparison between demographic and baseline characteristics of the two groups. Patients with AKI were older (61.13 vs. 56.52 years, *p* < 0.001) and had a higher heart rate, respiratory rate, and SBP (all *p* < 0.05) than those without AKI. They exhibited higher SOFA, SAPS II, and CCI scores as well as higher eGFR, WBC, RDW, bicarbonate, BUN, glucose, INR, and potassium levels. They also had a higher proportion of GCS <13 (27.79% vs. 21.01%, *p* = 0.009) than those without AKI. There was no significant unadjusted difference between the cohorts with respect to calcium exposure.

**Table 1:** Patients' characteristics.

**Variables**	**Total (*n =* 1,128)**	**Non-AKI (*n =* 671)**	**AKI (*n =* 457)**	**Statistics**	***p***
Age, year, Mean ± SD	58.39 ± 14.81	56.52 ± 14.29	61.13 ± 15.16	t = −5.18	<0.001
Gender, *n* (%)				χ^2^ = 0.032	0.857
Women	648 (57.45)	384 (57.23)	264 (57.77)		
Men	480 (42.55)	287 (42.77)	193 (42.23)		
Race, *n* (%)				χ^2^ = 0.152	0.927
Black	97 (8.60)	59 (8.79)	38 (8.32)		
Other	183 (16.22)	107 (15.95)	76 (16.63)		
White	848 (75.18)	505 (75.26)	343 (75.05)		
Heart rate, bpm, Mean ± SD	80.20 ± 17.34	78.58 ± 15.82	82.58 ± 19.12	t = −3.70	<0.001
Temperature, °C, Mean ± SD	36.78 ± 0.78	36.79 ± 0.74	36.77 ± 0.84	t = 0.45	0.650
Respiratory rate, bpm, Mean ± SD	17.48 ± 4.84	17.07 ± 4.56	18.07 ± 5.17	t = −3.35	<0.001
SBP, mmHg, Mean ± SD	133.12 ± 21.84	131.55 ± 20.47	135.43 ± 23.53	t = −2.86	0.004
DBP, mmHg, Mean ± SD	70.72 ± 15.08	70.31 ± 14.95	71.32 ± 15.25	t = −1.11	0.268
SOFA, score, M (Q_1_, Q_3_)	3.00 (1.00, 5.00)	2.00 (1.00, 4.00)	4.00 (2.00, 5.00)	Z = 8.746	<0.001
SAPS II, score, M (Q_1_, Q_3_)	28.00 (22.00, 37.00)	26.00 (22.00, 34.00)	31.00 (24.00, 41.00)	Z = 7.122	<0.001
GCS, *n* (%)				χ^2^ = 6.892	0.009
<13	268 (23.76)	141 (21.01)	127 (27.79)		
≥13	860 (76.24)	530 (78.99)	330 (72.21)		
CCI, score, M (Q_1_, Q_3_)	3.00 (1.00, 4.00)	2.00 (1.00, 4.00)	3.00 (1.00, 5.00)	Z = 4.274	<0.001
eGFR, ratio, Mean ± SD	93.18 ± 21.59	95.93 ± 20.67	89.16 ± 22.30	t = 5.23	<0.001
WBC, K/uL, M (Q_1_, Q_3_)	11.20 (8.50, 14.50)	10.70 (8.20, 13.70)	12.10 (9.10, 15.40)	Z = 5.053	<0.001
Hemoglobin, g/dL, Mean ± SD	12.12 ± 1.79	12.11 ± 1.70	12.14 ± 1.90	t = −0.27	0.786
Platelet count, K/uL, M (Q_1_, Q_3_)	222.00 (183.50, 278.50)	223.00 (187.00, 278.00)	221.00 (173.00, 279.00)	Z = −0.945	0.345
RDW, %, Mean ± SD	13.71 ± 1.46	13.58 ± 1.42	13.89 ± 1.49	t = −3.52	<0.001
Hematocrit, %, Mean ± SD	35.71 ± 5.07	35.62 ± 4.76	35.85 ± 5.50	t = −0.72	0.473
Bicarbonate, mEq/L, Mean ± SD	23.20 ± 3.27	23.37 ± 3.22	22.95 ± 3.33	t = 2.12	0.035
BUN, mg/dL, M (Q_1_, Q_3_)	12.00 (9.00, 16.00)	12.00 (9.00, 15.00)	14.00 (10.00, 17.00)	Z = 6.281	<0.001
Glucose, mg/dL, M (Q_1_, Q_3_)	131.00 (112.00, 156.00)	128.00 (109.00, 148.00)	141.00 (117.00, 175.00)	Z = 5.737	<0.001
INR, ratio, M (Q_1_, Q_3_)	1.10 (1.00, 1.20)	1.10 (1.00, 1.20)	1.10 (1.00, 1.20)	Z = 4.949	<0.001
Chloride, mEq/L, Mean ± SD	105.15 ± 4.56	105.13 ± 4.38	105.18 ± 4.82	t = −0.18	0.858
Sodium, mEq/L, Mean ± SD	138.72 ± 3.94	138.84 ± 3.66	138.55 ± 4.31	t = 1.16	0.244
Magnesium, mEq/L, Mean ± SD	1.85 ± 0.25	1.86 ± 0.25	1.84 ± 0.26	t = 1.45	0.146
Potassium, mEq/L, Mean ± SD	3.89 ± 0.51	3.86 ± 0.49	3.93 ± 0.55	t = −2.18	0.030
Aneurysm Clipping, *n* (%)				χ^2^ = 0.056	0.813
No	1,049 (93.00)	625 (93.14)	424 (92.78)		
Yes	79 (7.00)	46 (6.86)	33 (7.22)		
Aneurysm Embolization, *n* (%)				χ^2^ = 0.144	0.704
No	841 (74.56)	503 (74.96)	338 (73.96)		
Yes	287 (25.44)	168 (25.04)	119 (26.04)		
CCB, *n* (%)				χ^2^ = 1.825	0.177
No	283 (25.09)	178 (26.53)	105 (22.98)		
Yes	845 (74.91)	493 (73.47)	352 (77.02)		
ACE inhibitors, *n* (%)				χ^2^ = 1.523	0.217
No	918 (81.38)	554 (82.56)	364 (79.65)		
Yes	210 (18.62)	117 (17.44)	93 (20.35)		
Mannitol use, *n* (%)				χ^2^ = 13.667	<0.001
No	1,040 (92.20)	635 (94.63)	405 (88.62)		
Yes	88 (7.80)	36 (5.37)	52 (11.38)		
Osmolar contrast use, *n* (%)				χ^2^ = 0.174	0.677
No	1,115 (98.85)	664 (98.96)	451 (98.69)		
Yes	13 (1.15)	7 (1.04)	6 (1.31)		
Calcium, mEq/L, Mean ± SD	2.14 ± 0.18	2.15 ± 0.17	2.13 ± 0.19	t = 1.34	0.180
Calcium level, *n* (%)				χ^2^ = 5.905	0.052
<2.0709	343 (30.41)	187 (27.87)	156 (34.14)		
2.0709–2.2206	400 (35.46)	253 (37.70)	147 (32.17)		
≥2.2206	385 (34.13)	231 (34.43)	154 (33.70)		

T, *t*-test; Z, Wilcoxon–Mann–Whitney test; χ^2^, chi-squared test; SD, standard deviation; M, median; Q1, 1st quartile; Q3, 3rd quartile. AKI, acute kidney injury; SBP, systolic blood pressure; DBP, diastolic blood pressure; SOFA, sequential organ failure assessment score; SAPS II, simplified acute physiology score II; GCS, Glasgow Coma Scale; CCI, Charlson Comorbidity Index; eGFR, estimated glomerular filtration rate; WBC, white blood cell count; RDW, red blood cell distribution width; BUN, blood urea nitrogen; INR, international normalized ratio; CCB, calcium channel blockers; ACE, angiotensin-converting enzyme.

### Association between serum calcium level and the risk of AKI in SAH patients

**Figure 2:**
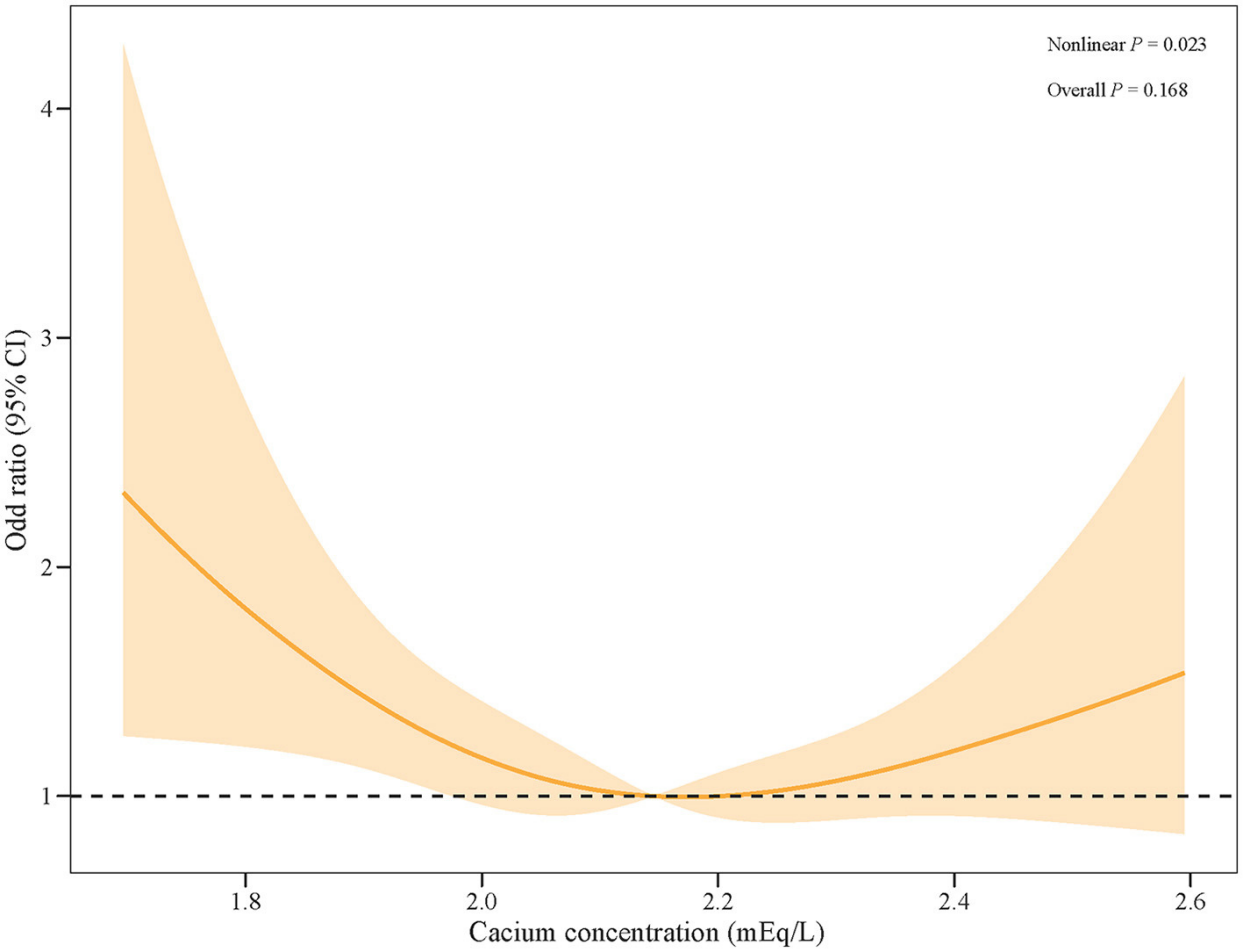
The RCS curve of the risk of AKI along with the change in serum calcium levels in patients with SAH.

The logistic regression models showed that with serum calcium level (2.0709–2.2206) as the reference, a low level of serum calcium was significantly associated with a high risk of AKI in patients with SAH, with an OR (95%CI) of 1.38 (1.01–1.89), after adjusting for age, WBC, SBP, heart rate, BUN, glucose, INR, CCI, and mannitol use, as shown in Table 2. Similarly, the RCS curve clearly shows that there was a significant non-linear association between serum calcium level and the risk of AKI in SAH patients (Figure 2).

**Table 2:** Association between serum calcium levels and the risk of AKI in SAH patients.

**Variables**	**Model 1**		**Model 2**	
	**OR (95%CI)**	***p***	**OR (95%CI)**	***p***
Calcium	0.63 (0.32–1.22)	0.168	0.55 (0.27–1.13)	0.104
**Calcium level**				
<2.0709	1.44 (1.07–1.93)	0.016	1.38 (1.01–1.89)	0.047
2.0709–2.2206	Ref		Ref	
≥2.2206	1.15 (0.86–1.53)	0.349	0.99 (0.73–1.35)	0.958

Ref, reference; OR, odd ratio; CI, confidence interval.

Model 1: univariate logistic regression model;

Model 2: adjustment for age, WBC, SBP, heart rate, BUN, glucose, INR, CCI, and mannitol use.

AKI, acute kidney injury; SAH, subarachnoid hemorrhage.

### Subgroup analyses stratified by age, GCS, drugs, and surgical methods

**Figure 3:**
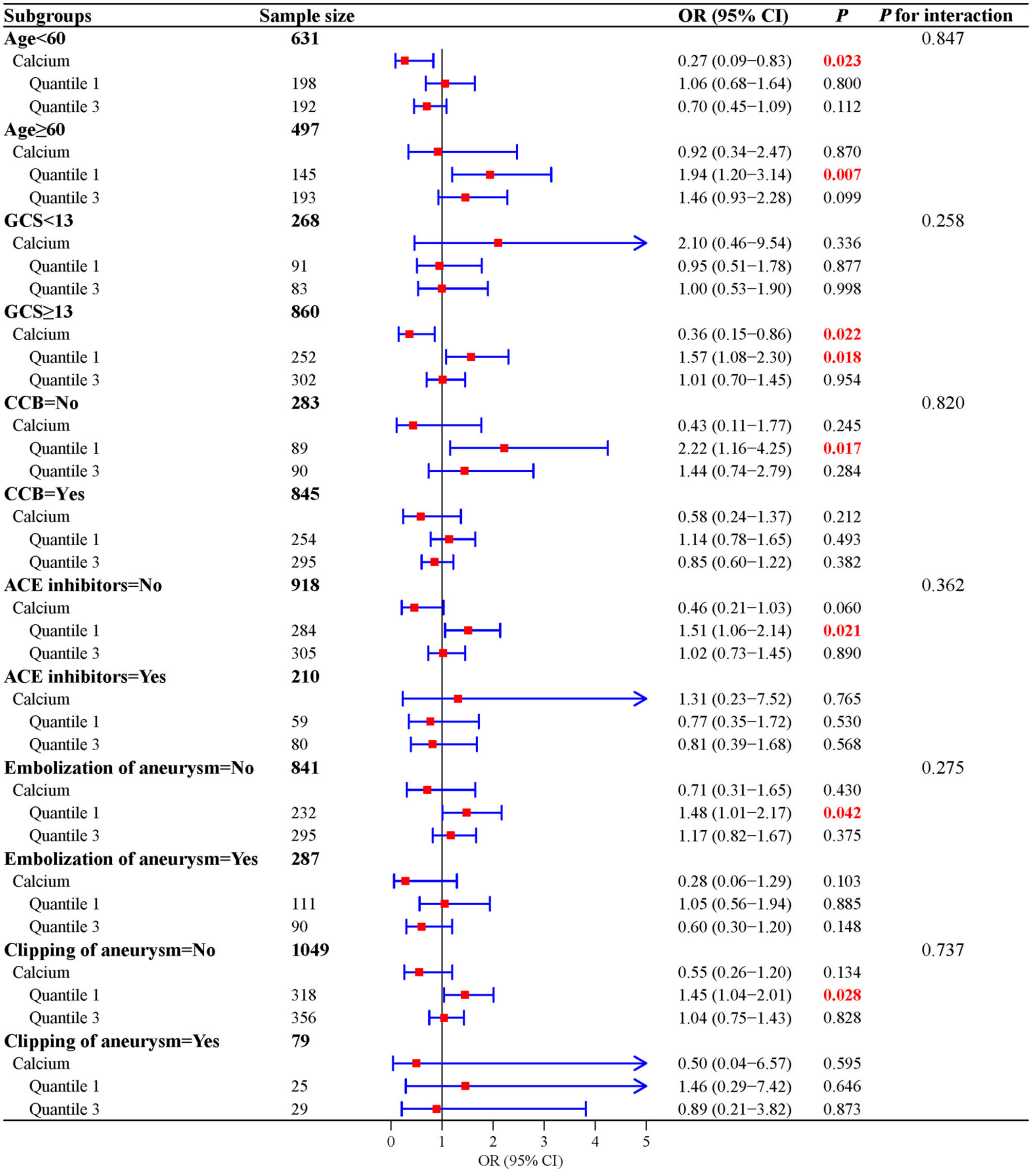
Subgroup analyses of the association between serum calcium levels and the risk of AKI.

To further explore the association, subgroup analyses were performed. The results suggested that a low level of calcium was significantly associated with a high risk of AKI in SAH patients who were aged ≥60 years (OR = 0.27, 95%CI: 0.09–0.83), who had a GCS score of ≥13 (OR = 1.57, 95%CI: 1.08–2.30), who did not use CCB (OR = 2.22, 95%CI: 1.16–4.25) and ACE inhibitors (OR = 1.51, 95%CI: 1.06–2.14), and who did not undergo aneurysm embolization (OR = 1.48, 95%CI: 1.01–2.17) and aneurysm clipping (OR = 1.45, 95%CI: 1.04–2.01). Detailed results are shown in Figure 3.

## Discussion

This study aimed to investigate the effect of serum calcium level on the risk of AKI in patients with SAH. We demonstrated that a low level of serum calcium was associated with a high risk of AKI in SAH patients after adjusting for potential confounding factors. Further subgroup analyses showed that a low level of calcium was significantly associated with a high risk of AKI in SAH patients who were aged ≥60 years, who had GCS score of ≥13, who did not use CCB and ACE inhibitors, and who did not undergo aneurysm embolization and aneurysm clipping.

Calcium is an essential mineral required for many physiological functions in the body and plays an important role in many biological processes, including enzymatic activity, cardiac contraction and relaxation, and the contraction of the vascular smooth muscle (19, 23, 30, 31). Although no study has explored the association between serum calcium level and the risk of AKI in patients with SAH, observational studies have reported that serum calcium levels are associated with a higher risk of intracerebral hemorrhage (ICH), hemorrhagic stroke, and SAH (32–34). A study by Morotti et al. (32) (*n* = 2,103) suggested that low calcium levels were independently associated with a higher baseline ICH volume. A retrospective cohort study (*n* = 67, Liu et al.) suggested that lower levels of serum calcium (≤ 2.15 mmol/L) were independently associated with the presence of cerebral microbleeds (CMBs) and deep CMBs in ischemic stroke patients with atrial fibrillation and/or rheumatic heart disease (33). Zhang et al. found that genetically predicted serum calcium and serum parathyroid hormone levels were associated with aneurysmal SAH (34). Recently, studies have reported a U-shaped association between serum calcium levels and the risk of AKI in hospitalized patients (14). Thongprayoon et al. (35) performed a single-center retrospective study of 1,779 hospitalized patients and suggested that the risk of AKI was increased not only in patients with an elevated serum calcium level of ≥9.0 mg/dL but also in those with a decreased serum calcium level of ≤ 7.9 mg/dL on admission. In addition, different studies have found that cardiovascular complications, such as low left ventricular ejection fraction, left ventricular volume load overload, and cardiac arrhythmia, which are related to low levels of serum calcium, may contribute to the development of AKI (19, 21–24). Based on the above research, which considers a potential U-shaped relationship between calcium and the risk of AKI, we established a reference level for serum calcium concentration at 2.0709–2.2206 mEq/L. Our results suggest that a low level of serum calcium within the range of 2.0709–2.2206 mEq/L is significantly associated with a high risk of AKI in patients with SAH. This information may be valuable for the management of AKI risk and an improved prognosis in patients with SAH.

Our study also focused on the association between serum calcium levels and specific subgroups, such as age, GCS, drugs, and surgical methods. A significant positive association between serum calcium level and the risk of AKI was observed in SAH patients aged ≥60 years old. The ICU population tends to be older with a high incidence of AKI in those older than 65 years (36). In older adult patients, the likelihood of developing AKI increases due to several factors: they may have multiple comorbid conditions, experience age-related hemodynamic changes (37), use more medications (38), and undergo intrinsic kidney changes as they age. Calcium homeostasis may influence the risk of AKI by regulating these pathways. GCS is one of the most reliable evaluation indicators used for evaluating the level of consciousness in neurosurgical patients, especially in patients with SAH (39). A score of 13–15 indicates a mild disturbance of consciousness. Our study showed that a low level of serum calcium was significantly associated with a high risk of AKI in SAH patients who had a GCS score of ≥13. Ionized calcium is an essential cofactor in the coagulation cascade and platelet aggregation, and hypocalcemia may contribute to the progression of intracranial bleeding. According to a previous study, among patients with isolated severe traumatic brain injury, mild hypocalcemia at admission is associated with better neurological status at hospital discharge. This association is especially noted among patients with a GCS score of >8 at admission (40). However, our results suggested that low levels of calcium could predict a higher probability of AKI when SAH patients have a mild disturbance of consciousness. SAH may trigger nerve damage. A prolonged electrolyte imbalance, such as metabolic calcium disorder, can significantly affect the outcomes of neurological injuries (41). In addition, SAH-related headaches are associated with serum calcium concentration (42). Therefore, it is important to monitor serum calcium levels in patients with different characteristics of SAH to reduce the potential risk of AKI. In addition, a possible reason why a low level of calcium was associated with a high risk of AKI only in patients who did not receive CCB or ACE inhibitor therapy might be that these antihypertensive agents can reduce the concentration of cytosolic calcium and prevent some of the pathogenic mechanisms involved in kidney injury, thereby reducing chronic kidney disease (CKD) progression (43, 44). In addition, aneurysm embolization and aneurysm clipping can largely prevent and inhibit intracranial hemorrhage, which have been widely used in the treatment of SAH (45, 46). Our results showed that, in patients with SAH who neither used CCB and ACE inhibitors nor underwent aneurysm embolization and aneurysm clipping, a low level of serum calcium was significantly associated with a high risk of AKI. This finding may be due to the therapeutic effect of drugs and surgery on the condition, which weakened the correlation between low levels of serum calcium and a high risk of AKI in SAH patients.

Several factors may explain why a low level of serum calcium is associated with a high risk of AKI in patients with SAH. Serum calcium plays an important role in platelet function and the coagulation cascade (47, 48). In mouse models, Erreger et al. (49) suggested that protease-activated receptor 4 (PAR4) contributes to the pathology observed in AKI. Both protease-activated receptor 1 (PAR1) and PAR4 are G-protein-coupled receptors activated by thrombin, and in platelets, response to thrombin PAR4 contributes to the predominant formation of procoagulant microparticles, the augmentation of fibrin deposition, and the initiation of platelet-stimulated inflammation (49). PAR4 signal via Gq mobilizes intracellular calcium and drives platelet function (50). It has been reported that PAR1 activation after SAH increases microvascular permeability (51). Therefore, investigating whether PAR4 takes part in the mechanism by which serum calcium concentration influences the risk of AKI in patients with SAH may be an interesting research direction in the future. Meanwhile, the characteristics of AKI are the activation of the intra-renal hemostatic and inflammatory processes. Platelets are present in high numbers in the circulation and are important acute modulators of inflammation and hemostasis, interacting with endothelial cells and leukocytes at the sites of acute injury. Diminished control of platelet reactivity by endothelial cells and/or an increased release of platelet-activating mediators can lead to uncontrolled platelet activation in AKI (52). Calcium ion is a key endothelial cytoplasm that regulates paracellular permeability in the blood–brain barrier (53). In various kidney diseases, elevated vascular permeability exacerbates renal structural and functional disorders through pathways that mainly act on vascular endothelial cadherin to modulate adherens junctions of endothelial cells, thereby augmenting vascular permeability via the paracellular pathway. In this study, we speculated that, among SAH patients, the concentration of serum calcium may also be associated with the subsequent development of AKI through its influence on vascular permeability by regulating the paracellular pathway.

This study is the first to demonstrate that a low level of serum calcium is significantly associated with a high risk of AKI in patients with SAH. Serum calcium levels were easily detected. Physicians can further identify high-risk patients or allow the early detection of AKI by assessing serum calcium levels at admission. This may provide a window of opportunity for early therapeutic intervention in patients with SAH. The present study also has several limitations. First, this study was a retrospective cohort study, which inevitably had a certain selection bias and reporting bias. Second, due to the limitation of the MIMIC database, there could have been some unmeasured confounding factors that might have affected the results of the association between serum calcium levels and AKI, such as the location of the aneurysm rupture, cerebral blood flow changes, hemorrhage volume, and pathological features of SAH. Finally, this study only analyzed the association between the baseline calcium level and the risk of AKI in patients with SAH but did not consider the effect of changes in the calcium levels on outcomes.

## Conclusion

The results of our study indicated that a low level of calcium is a significant predictor of developing AKI in SAH patients admitted to the ICU, which provides a certain reference for risk stratification and the management of patients with SAH.

## Data Availability

Publicly available datasets were analyzed in this study. This data can be found here, MIMIC-III and MIMIC-IV databases, https://mimic.physionet.org/iii/ and https://mimic.physionet.org/iv/.
