# Supplementary material for: Association between serum calcium level and the risk of acute kidney injury in ICU patients with subarachnoid hemorrhage: a retrospective cohort study
Source: Front Neurol. 2024 Dec 11;15:1433653. doi: 10.3389/fneur.2024.1433653 (PMC11670206; doi:10.3389/fneur.2024.1433653)
Supplement: Supplementary file 1 [file Table_1.DOCX]

Table S1. Sensitivity analyses for data before and after imputation

| Variables | After imputation | Before imputation | Statistics | *P* |
| --- | --- | --- | --- | --- |
| Race, n (%) |  |  | χ^2^=0.059 | 0.971 |
| Black | 97 (8.60) | 88 (8.88) |  |  |
| Other | 183 (16.22) | 159 (16.04) |  |  |
| White | 848 (75.18) | 744 (75.08) |  |  |
| Temperature, Deg. C, Mean ± SD | 36.78 ± 0.78 | 36.78 ± 0.78 | t=0.01 | 0.991 |
| Respiratory rate, bpm, Mean ± SD | 17.48 ± 4.84 | 17.44 ± 4.79 | t=0.21 | 0.834 |
| DBP, mmHg, Mean ± SD | 70.72 ± 15.08 | 70.71 ± 15.09 | t=0.01 | 0.988 |
| eGFR, ratio, Mean ± SD | 93.18 ± 21.59 | 93.16 ± 21.65 | t=0.03 | 0.978 |
| WBC, K/uL, M (Q_1_, Q_3_) | 11.20 (8.50, 14.50) | 11.20 (8.50, 14.50) | Z=-0.018 | 0.986 |
| Hemoglobin, g/dL, Mean ± SD | 12.12 ± 1.79 | 12.11 ± 1.79 | t=0.04 | 0.968 |
| Platelet count, K/uL, M (Q_1_, Q_3_) | 222.00 (183.50, 278.50) | 222.00 (183.00, 279.00) | Z=-0.022 | 0.982 |
| RDW, %, Mean ± SD | 13.71 ± 1.46 | 13.70 ± 1.45 | t=0.11 | 0.914 |
| Hematocrit, g/dL, Mean ± SD | 35.71 ± 5.07 | 35.70 ± 5.07 | t=0.05 | 0.958 |
| Bicarbonate, mEq/L, Mean ± SD | 23.20 ± 3.27 | 23.20 ± 3.28 | t=0.00 | 1.000 |
| BUN, mg/dL, M (Q_1_, Q_3_) | 12.00 (9.00, 16.00) | 12.00 (10.00, 16.00) | Z=0.108 | 0.914 |
| Glucose, mg/dL, M (Q_1_, Q_3_) | 131.00 (112.00, 156.00) | 131.00 (112.00, 156.00) | Z=0.093 | 0.926 |
| INR, ratio, Mean ± SD | 1.16 ± 0.31 | 1.18 ± 0.31 | t=-1.16 | 0.245 |
| Chloride, mEq/L, Mean ± SD | 105.15 ± 4.56 | 105.15 ± 4.57 | t=-0.01 | 0.990 |
| Sodium, mEq/L, Mean ± SD | 138.72 ± 3.94 | 138.73 ± 3.95 | t=-0.05 | 0.964 |
| Magnesium, mEq/L, Mean ± SD | 1.85 ± 0.25 | 1.85 ± 0.26 | t=0.19 | 0.846 |
| Potassium, mEq/L, Mean ± SD | 3.89 ± 0.51 | 3.89 ± 0.52 | t=0.05 | 0.960 |

T: T-test, Z: Wilcoxon-Mann-Whitney test, χ^2^: Chi-square test, SD: standard deviation, M: median, Q1:1st quartile, Q3:3st quartile.

DBP: diastolic blood pressure; eGFR: estimated glomerular filtration rate; WBC: white blood cell count; RDW: red blood cell distribution width; BUN: blood urea nitrogen; INR: international normalized ratio.

Table S2. Screening the confounding factors of affecting the occurrence of AKI

| Characteristics | OR (95% CI) | *P* |
| --- | --- | --- |
| Glucose | 1.01 (1.01-1.01) | 0.001 |
| BUN | 1.03 (1.01-1.05) | 0.009 |
| INR | 2.51 (1.42-4.44) | 0.002 |
| WBC | 1.06 (1.03-1.09) | <0.001 |
| Age | 1.02 (1.01-1.03) | 0.004 |
| SBP | 1.01 (1.01-1.01) | 0.019 |
| Heart rate | 1.01 (1.00-1.02) | 0.053 |
| CCI | 1.06 (0.99-1.13) | 0.090 |
| Mannitol use |  |  |
| No | Ref |  |
| Yes | 2.00 (1.25-3.21) | 0.004 |

OR: odd ratio, CI: confidence interval.

BUN: blood urea nitrogen; INR: international normalized ratio; WBC: white blood cell count; SBP: systolic blood pressure; CCI: charlson comorbidity index.
